# Development of expression vectors based on pepino mosaic virus

**DOI:** 10.1186/1746-4811-7-6

**Published:** 2011-03-11

**Authors:** Raquel N Sempere, Pedro Gómez, Verónica Truniger, Miguel A Aranda

**Affiliations:** 1 Departamento de Biología del Estrés y Patología Vegetal, Centro de Edafología y Biología Aplicada del Segura (CEBAS)- CSIC, PO Box 164, 30100 Espinardo, Murcia, Spain; 2 Bioprodin SL, Edificio CEEIM, Campus de Espinardo s/n, 30100 Espinardo, Murcia, Spain; 3 Department of Zoology, Oxford University, Oxford OX1 3PS, UK

## Abstract

**Background:**

Plant viruses are useful expression vectors because they can mount systemic infections allowing large amounts of recombinant protein to be produced rapidly in differentiated plant tissues. Pepino mosaic virus (PepMV) (genus *Potexvirus*, family *Flexiviridae*), a widespread plant virus, is a promising candidate expression vector for plants because of its high level of accumulation in its hosts and the absence of severe infection symptoms. We report here the construction of a stable and efficient expression vector for plants based on PepMV.

**Results:**

Agroinfectious clones were produced from two different PepMV genotypes (European and Chilean), and these were able to initiate typical PepMV infections. We explored several strategies for vector development including coat protein (CP) replacement, duplication of the CP subgenomic promoter (SGP) and the creation of a fusion protein using the foot-and-mouth disease virus (FMDV) 2A catalytic peptide. We found that CP replacement vectors were unable to move systemically and that vectors with duplicated SGPs (even heterologous SGPs) suffered from significant transgene instability. The fusion protein incorporating the FMDV 2A catalytic peptide gave by far the best results, maintaining stability through serial passages and allowing the accumulation of GFP to 0.2-0.4 g per kg of leaf tissue. The possible use of PepMV as a virus-induced gene silencing vector to study gene function was also demonstrated. Protocols for the use of this vector are described.

**Conclusions:**

A stable PepMV vector was generated by expressing the transgene as a CP fusion using the sequence encoding the foot-and-mouth disease virus (FMDV) 2A catalytic peptide to separate them. We have generated a novel tool for the expression of recombinant proteins in plants and for the functional analysis of virus and plant genes. Our experiments have also highlighted virus requirements for replication in single cells as well as intercellular and long-distance movement.

## Introduction

Pepino mosaic virus (PepMV) is a widespread plant virus that causes a major disease of tomato crops worldwide [1-11]. PepMV belongs to the genus *Potexvirus *(family *Flexiviridae*) and, like other members of this genus, has virions that are non-enveloped flexuous rods 508 nm in length [12]. The PepMV genome is a single-stranded RNA molecule approximately 6.4 kb in length, comprising five open reading frames (ORFs) flanked by 5' and 3' untranslated regions (UTRs) with a 5'-cap and a 3' poly-A tail. The ORFs encode a 164-kDa RNA-dependent RNA polymerase (RdRp); three triple gene block (TGB) proteins named TGBp1 (26 kDa), TGBp2 (14 kDa) and TGBp3 (9 kDa); and a 25-kDa coat protein (CP) [1,13]. Gene expression is thought to be similar to that in other potexviruses, *i.e. *the viral replicase is expressed from the genomic RNA (gRNA) whereas the TGB proteins are expressed from subgenomic RNAs (sgRNAs) 1 and 2, and the CP from sgRNA3 [1,13-15]. However, none of the above has been established empirically for PepMV, and little is known about its molecular interactions with host cells.

PepMV is a promising candidate expression vector for plants because of its high level of accumulation in its hosts and the absence of severe infection symptoms for some isolates, factors that have already led to the development of other potexvirus vectors [16,17]. Plant viruses are useful expression vectors because they can mount systemic infections allowing large amounts of recombinant protein to be produced rapidly in differentiated plant tissues, which makes them particularly suitable for the production of subunit vaccines [18-22]. However, they are also useful for basic research, *e.g*. as virus-induced gene silencing (VIGS) tools for reverse genetic studies of gene function [23-29] or to investigate the molecular basis of plant-virus interactions [16,30,31].

Many different approaches have been used to convert viruses into expression vectors, including gene replacement, gene insertion, epitope presentation and complementation [16,17,32-36]. Vectors based on tobacco mosaic virus (TMV), potato virus X (PVX), alfalfa mosaic virus (AMV), cucumber mosaic virus (CMV), and cowpea mosaic virus (CPMV) have been engineered as expression vectors and used to produce functional recombinant proteins including vaccines, antigens and enzymes [32,34,37-45]. Early vectors suffered from limitations such as instability and low yields, but this has been addressed by the genetic modification of vector sequences and by delivering virus vectors into plant cells using *Agrobacterium tumefaciens *[39,40].

Here we describe the development of a PepMV-based vector capable of systemic reporter gene expression in *Nicotiana benthamiana *plants. We explored different strategies to achieve stable transgene expression, including CP gene substitution, duplication of the CP subgenomic promoter (SGP) and the creation of a translational gene fusion. A stable PepMV vector was generated by expressing the transgene as a CP fusion using the sequence encoding the foot-and-mouth disease virus (FMDV) 2A catalytic peptide to separate them. We have generated a novel tool for the expression of recombinant proteins in plants, for the analysis of PepMV-plant interactions and for gene function studies. Our experiments have also highlighted virus requirements for replication in single cells as well as intercellular and long-distance movement.

## Results

### Agroinfectious PepMV clones

Isolates PepMV-Sp13 (European tomato genotype) and PepMV-Ps5 (Chilean genotype) [3,13] were used to produce agroinfectious clones [46]. Full-length cDNAs generated by RT-PCR were inserted into vector pBIN61 [47] between the cauliflower mosaic virus (CaMV) 35*S *promoter and the *nos *terminator, generating plasmids pBPepXL6 (for PepMV-Sp13) and pBPepPs5 (for PepMV-Ps5). Agroinfiltration of *N. benthamiana *leaves with *A. tumefaciens *containing either of the plasmids revealed that both clones were infectious, and very efficient (100% of plants were infected in each experiment). Agroinoculated plants developed symptoms identical to those induced by the original virus isolates, including mild mosaics, leaf bubbling and stunting. Plants agroinfiltrated with pBPepXL6 showed mild symptoms by 5 days post inoculation (dpi), whereas pBPepPs5 induced more severe symptoms, although these did not appear until 10 dpi. In both cases, infection was readily transmitted to other plants via sap. Dot-blots of total RNA extracted from systemically infected leaves from all the inoculated plants generated a strong hybridization signal when probed with a PepMV replicase sequence, confirming replication and movement of the virus (data not shown). We elected to develop pBPepXL6 as a vector because infection was faster, but accompanied by milder symptoms.

### Deletion or replacement of the PepMV coat protein gene

**Figure 1:**
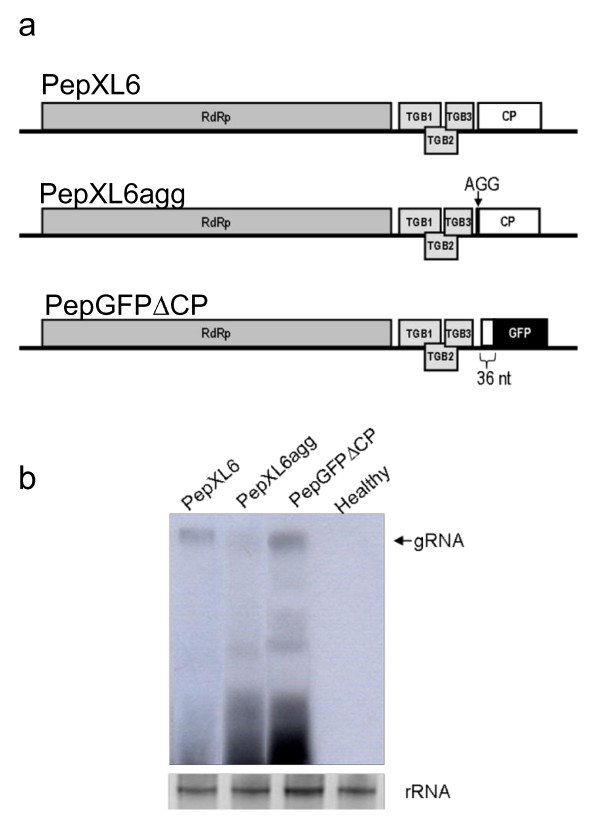
**Multiplication of PepMV CP-mutants in *N. benthamiana *protoplasts**. (a) Schematic representation of PepXL6 and its derived CP mutants (PepXL6agg and PepGFPΔCP). RdRp-RNA-dependent RNA polymerase ORF; TG1, TGB2 and TGB3-triple gene block ORFs; CP-coat protein ORF. (b) Northern blot of total RNA prepared from *N. benthamiana *protoplasts 24 h post inoculation, hybridized with a replicase-specific RNA probe. Ethidium bromide-stained rRNA is shown (bottom panels).

As a first step in vector development, we produced PepMV clones in which the CP gene was either mutated or replaced (Figure 1a). In PepXL6agg the first AUG codon was mutated to abolish CP expression, whereas in PepGFPΔCP most of the CP gene was replaced with the gene for green fluorescent protein (GFP) to create an in-frame fusion (Figure 1a). We produced two sets of three constructs (PepXL6agg, PepGFPΔCP and wild-type PepXL6), one set retaining the CaMV 35*S *promoter and *nos *terminator for expression *in planta*, and the other using the T7 promoter for run-off *in vitro *transcription. Northern blots of total RNA extracted from *N. benthamiana *protoplasts transfected with *in vitro *transcribed viral RNA showed that virus replication efficiency was construct-dependant, with PepGFPΔCP accumulating to similar levels as the wild type, but PepXL6agg accumulating in reproducibly lower amounts (Figure 1b). CP expression therefore does not appear necessary for PepMV replication, but the presence of a translatable ORF in the place of the CP ORF appears to promote virus accumulation at the single-cell level.

**Figure 2:**
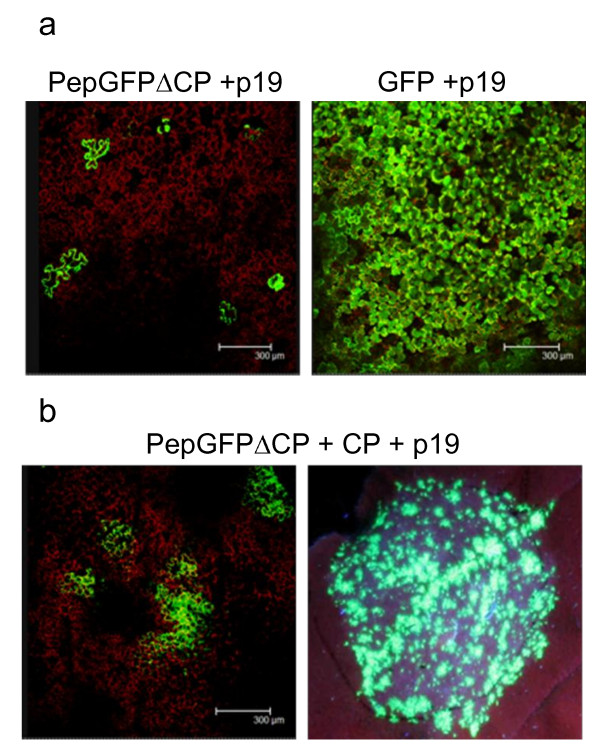
**Effect of CP deletion on PepMV multiplication in agroinfiltrated *N. benthamiana *tissues, with and without CP *trans *complementation**. (a) Leaves agroinfiltrated with pBPepGFPΔCP (left) and pBGFP (rigth) at 6 days post inoculation (dpi). (b) Leaves co-agroinfiltrated with pBPepGFPΔCP and the construct for CP expression, at 6 dpi (left) and 10 dpi (right). The agroinfiltration assays were carried out in the presence of pB19. Photographs at 6 dpi were taken by confocal laser scanning microscopy and the photograph at 10 dpi was taken under illumination with a handheld UV lamp.

Next, we agroinfiltrated *N. benthamiana *leaves with PepGFPΔCP in the presence of pBp19 (expressing the silencing suppressor P19) to avoid any time-dependant decline in GFP fluorescence [48]. GFP was typically observed in single epidermal and mesophyll cells (Figure 2a, left). Groups of three fluorescent cells were observed occasionally, but the fluorescence did not move to adjacent cells. Importantly, the number of cells expressing GFP in the area agroinfiltrated with pBPepGFPΔCP was very low compared to the control construct, pBGFP (Figure 2a, right). These results confirmed that although the CP is unnecessary for PepMV replication in single cells, it is required for virus movement, as shown for other potexviruses [49-51].

We carried out additional agroinfiltration experiments to determine whether CP provided in *trans *could complement PepGFPΔCP and restore intercellular movement. This was achieved by co-infiltrating *N. benthamiana *leaves with pBPepGFPΔCP and pBINCPep, containing the CP gene. Confocal laser scanning microscopy revealed that ~90% of the GFP foci in co-infiltrated leaves consisted of multiple (usually more than nine) cells (Figure 2b), confirming that PepMV CP movement functions could be provided in *trans*.

### PepMV vectors with a duplicated subgenomic mRNA promoter

**Figure 3:**
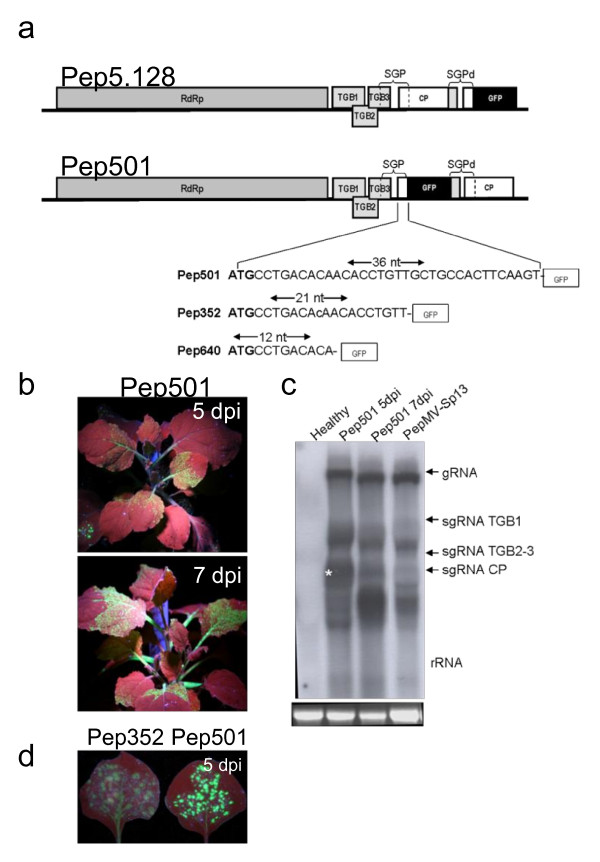
**PepMV vectors containing a duplicated subgenomic mRNA promoter**. (a) In Pep5.128, the GFP gene was positioned downstream of the CP gene, whereas it was positioned upstream of the CP gene in Pep501. The duplicated SGP spanned positions -76 to +36 relative to the CP start codon. Pep352 and Pep640 include successive deletions at the 3' end of the putative CP SGP. SGP-CP subgenomic mRNA promoter; SGPd-duplicated CP subgenomic mRNA promoter. (b) Green fluorescence visible in systemically-infected *N. benthamiana *leaves from plants agroinfiltrated with pBPep501 at 5 and 7 dpi. (c) Northern blot of leaf tissue hybridized with a CP-specific RNA probe to determine Pep501 stability. Total RNA was prepared from systemically-infected leaves at 5 and 7 dpi. The band corresponding to the GFP-CP sgRNA is marked with star. Ethidium bromide-stained rRNA is shown (bottom panels). (d) *N. benthamiana *leaves infiltrated with pBPep352 or pBPep501 vectors, showing fluorescence emitted under UV illumination with a handheld UV lamp. Photographs were taken at 5 or 7 dpi.

We next designed two full-length PepMV-GFP vectors, each carrying a duplicated CP subgenomic mRNA promoter (SGP). In Pep5.128, the GFP gene was positioned downstream the CP gene, whereas in Pep501 it was positioned upstream (Figure 3a). The duplicated SGP began 76 bp upstream of the CP start codon and ended 36 bp downstream, with the upstream sequence containing the potexvirus-specific octanucleotide 5'-GTTAAGTTT-3' [52,53]. The corresponding cDNAs were inserted into pBIN61 between the CaMV 35*S *promoter and *nos *terminator as above, resulting in constructs pBPep501 and pBPep5.128. *N. benthamiana *leaves were agroinfiltrated with these plasmids in the presence of pBp19 and GFP expression was monitored from 3-15 dpi. In each case, fluorescence was detected in the agroinfiltrated leaves and systemically by 5 dpi, confirming that the mutants were infectious and capable of intercellular movement (Figure 3b). However, virus spreading could only be monitored up to 10 dpi because GFP fluorescence fell below the detection threshold at this time. Northern blots of total RNA extracted from infected plants (agroinfiltrated and systemically-infected leaves) at different time-points revealed a signal corresponding to the predicted size of GFP-CP sgRNA by 5 dpi (Figure 3b, lane 2, marked with star) but this band was no longer visible by 7 dpi, another band matching the expected size of the wild type CP sgRNA was observed (Figure 3c, lane 3). This suggested that the GFP transgene had been excised from the vector genome by recombination during the course of the infection.

In an effort to improve stability, we attempted to reduce the size of the CP SGP by eliminating sequences from the 3' end (Pep352 contained 21 bp of the CP gene and Pep640 contained 12 bp, as shown in Figure 3a). The corresponding cDNAs were inserted into pBIN61 as described above and introduced into *N. benthamiana *leaves by agroinfiltration in the presence of pBp19. No fluorescence was observed in plants inoculated with Pep640 (data not shown), but fluorescence was detected in plants inoculated with Pep352 by 4 dpi both in agroinfiltrated and systemically-infected leaves. However, fluorescence was 55% less intense than in plants inoculated with Pep501, and began to decline by 7 dpi, suggesting even greater instability (Figure 3d).

### PepMV vectors with a heterologous CP subgenomic mRNA promoter

**Figure 4:**
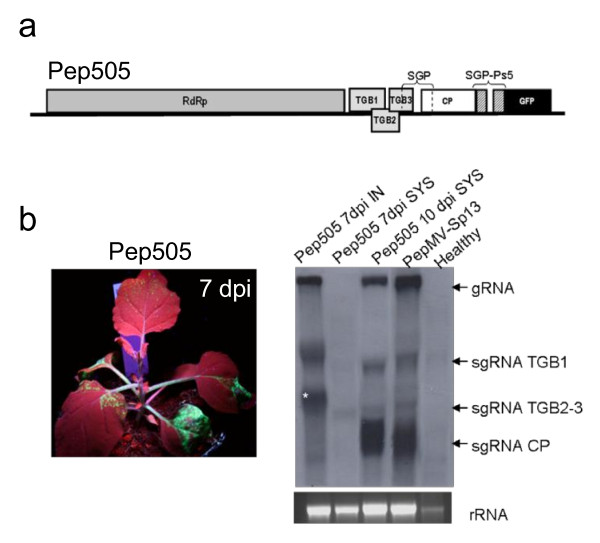
**PepMV vectors with a heterologous CP subgenomic mRNA promoter**. (a) Schematic representation of Pep505. SGP-Ps5 corresponds to the PepMV Ps5 (Chilean genotype) CP subgenomic mRNA promoter. (b) Fluorescence in systemically-infected leaves of *N. benthamiana *plants agroinfiltrated with pBPep505. Plants were photographed under illumination with a handheld UV lamp at 7 dpi. (c) Northern blot of total RNA from leaf tissues hybridized with a CP-specific RNA probe to determine Pep505 stability. Total RNA was prepared from agroinfiltrated (IN) and systemically-infected (SYS) leaves at 7 and 10 dpi. The band corresponding to the CP-GFP sgRNA is marked with star. Ethidium bromide-stained rRNA is shown (bottom panels).

To reduce the likelihood of excision by recombination, we constructed a vector based on PepMV-Sp13 that contained the GFP from the heterologous CP SGP of PepMV-Ps5. In this vector, named Pep505, the GFP gene was placed downstream of the CP gene (Figure 4a). Pep505 was capable of replication and systemic infection in *N. benthamiana *plants but was less efficient than Pep501. Systemic GFP fluorescence was observed by 5 dpi but had begun to decline in upper leaves by 7 dpi (Figure 4b). Northern blots of total RNA from infected plants (agroinfiltrated and systemically-infected leaves) at different time-points were hybridized with a probe specific for the CP gene (Figure 4c), showing the presence of a band of the predicted size for the CP-GFP sgRNA at 7 dpi (Figure 4c, lane 1, marked with star). In systemically-infected leaves, however, this signal began to fade between 7 and 10 dpi, and was replaced with a signal matching the predicted size of the wild-type CP gene. The heterologous sequence therefore did not improve vector stability.

### PepMV vectors including the FMDV 2A catalytic peptide sequence

**Figure 5:**
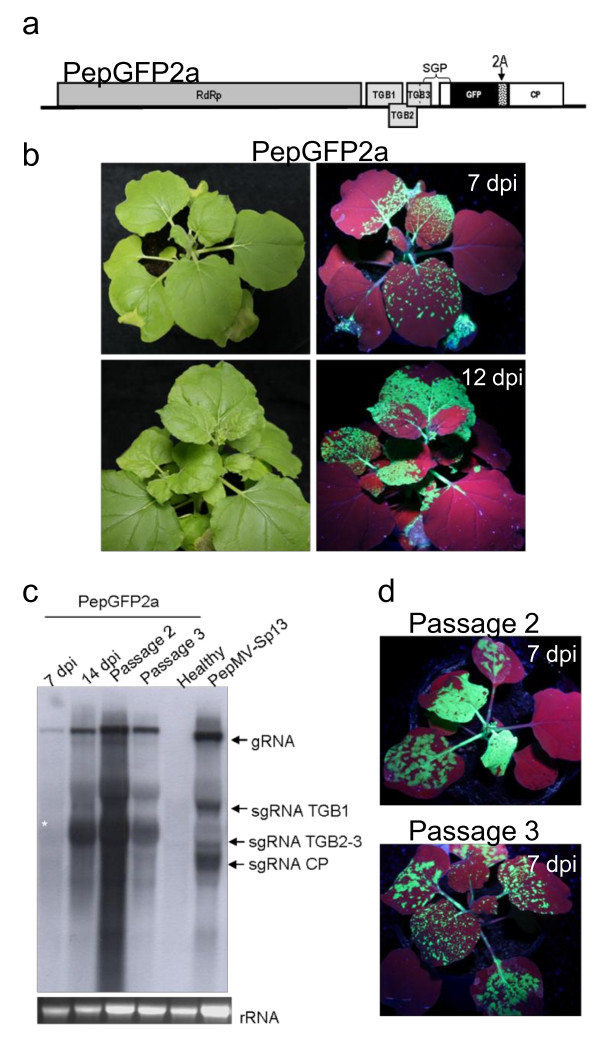
**PepMV vector including a GFP:CP fusion separated by the FMDV 2A catalytic peptide sequence**. (a) Schematic representation of PepGFP2a. The position of the 2A sequence insertion is indicated. (b) Fluorescence in systemically infected *N. benthamiana *leaves from plants agroinfiltrated with pBPepGFP2a. (c) Northern blot of leaf tissue hybridized with a CP-specific RNA probe to determine PepGFP2a stability. Total RNA was prepared from agroinfiltrated and systemically-infected leaves showing fluorescence at 7 and 14 dpi, and from plants mechanically inoculated with PepGFP2a at 7 dpi after the second and third passages. The band corresponding to the GFP-CP sgRNA is marked with star. Ethidium bromide-stained rRNA is shown (bottom panels). (d) GFP expression after second and third passage of PepGFP2a. Plants were photographed under white and UV light at 7 and 12 dpi.

In order to avoid sequence duplication, we explored an alternative expression strategy based on a CP fusion that included the FMDV 2A catalytic peptide sequence [54], resulting in vector pBPepGFP2a (Figure 5a). Following agroinfiltration, green fluorescent spots visible to the naked eye when leaves were illuminated with a handheld UV lamp appeared by 3-4 dpi. Subsequent long-distance movement of the virus to developing leaves led to the appearance of intense green fluorescence in systemically infected leaves 7-10 dpi (Figure 5b). The movement of PepGFP2a was therefore slower than Pep501, where systemic fluorescence was visible 5-6 dpi (Figure 3b). After 12 dpi, almost the complete plant expressed fluorescence, including aerial and subterranean parts. Mild or no visible symptoms of infection were observed during the course of the experiment. Northern blots of total RNAs extracted from fluorescent leaves 3-16 dpi revealed a signal corresponding in size to the CP2aGFP sgRNA (Figure 5c, lanes 1 and 2, marked with star).

Because PepGFP2a was the most promising candidate vector so far, we analyzed the stability of the GFP gene during serial passages, noting that GFP fluorescence was visible for several weeks in plants that were agroinfiltrated directly. We found that GFP fluorescence was maintained for up to four passages (Figure 5d), and Northern blots carried out 7 days after each passage confirmed the structural integrity of the virus (Figure 5c). During the third passage, at 12 dpi, GFP fluorescence began to decline in the upper leaves of the inoculated plants; Northern blots of total RNAs extracted from these leaves revealed a signal corresponding to the predicted size of the wild type CP sgRNA (data not shown), indicating that the vectors did eventually lose their integrity.

### Expression of GFP from PepGFP2a

**Figure 6:**
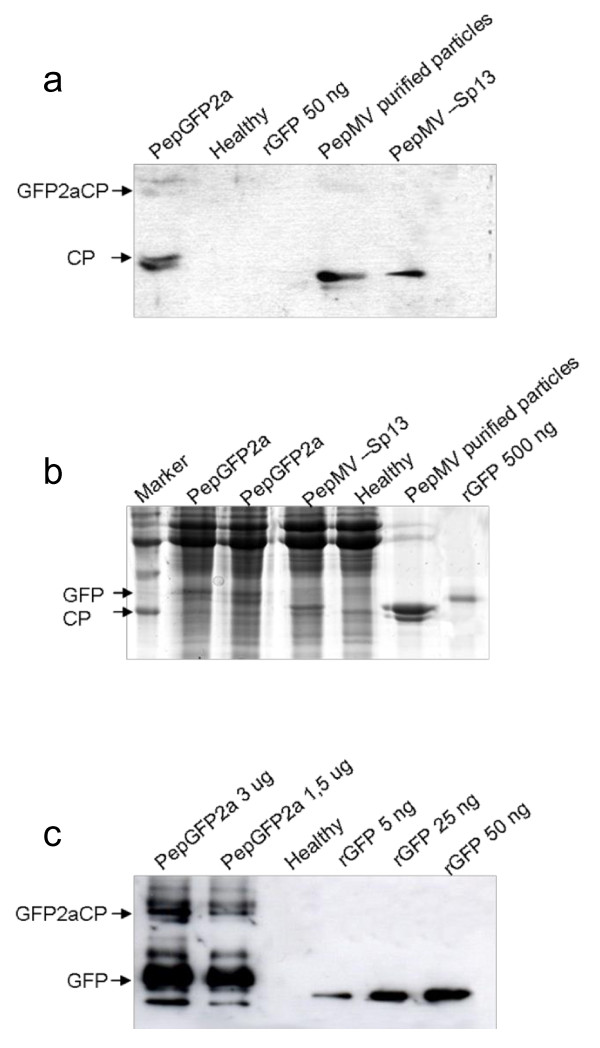
**Western blot analysis of total proteins extracted from systemically-infected leaves agroinfiltrated with PepGFP2a**. Soluble leaf proteins were separated by 15% SDS PAGE, followed by western blotting using an antibody against (a) the CP or (c) GFP. (b) Gel stained with Coomasie brilliant blue.

Protein extracts from systemically-infected leaves by 10 dpi were analyzed by western blot using a CP-specific antibody to determine whether the fusion protein was correctly processed. This revealed the presence of both a fusion protein (GFP2aCP) and a free CP, as well as a third signal representing a protein slightly larger than the free CP (Figure 6a); possibly, this third band corresponds to GFP plus the 12 immunorreactive amino-terminal amino acids of the CP fused to GFP due to the way the construct was designed.

The levels of GFP expression in plants agroinfiltrated with PepGFP2a were then investigated by SDS-PAGE and western blot. Total protein extracted from systemically-infected leaves was separated by SDS-PAGE, and a strong band migrating in the position expected for free GFP was observed in the Coomasie-stained gel (Figure 6b, lanes 2 and 3). This band was missing from the total protein extract prepared from leaves systemically infected with wild-type PepMV. The identity of this band was confirmed by western blot using an anti-GFP antibody, revealing that fluorescent leaves contained significant amounts of free GFP protein (Figure 6c). Relatively little of the GFP2aCP fusion protein was detected, indicating that cleavage by the 2A peptide was nearly complete. Based on these data, plus the results of quantitative ELISA experiments (data not shown), we estimated that expression levels of 0.2-0.4 g/kg leaf tissue could be achieved in systemically-infected leaves.

### A VIGS vector based on PepMV

**Figure 7:**
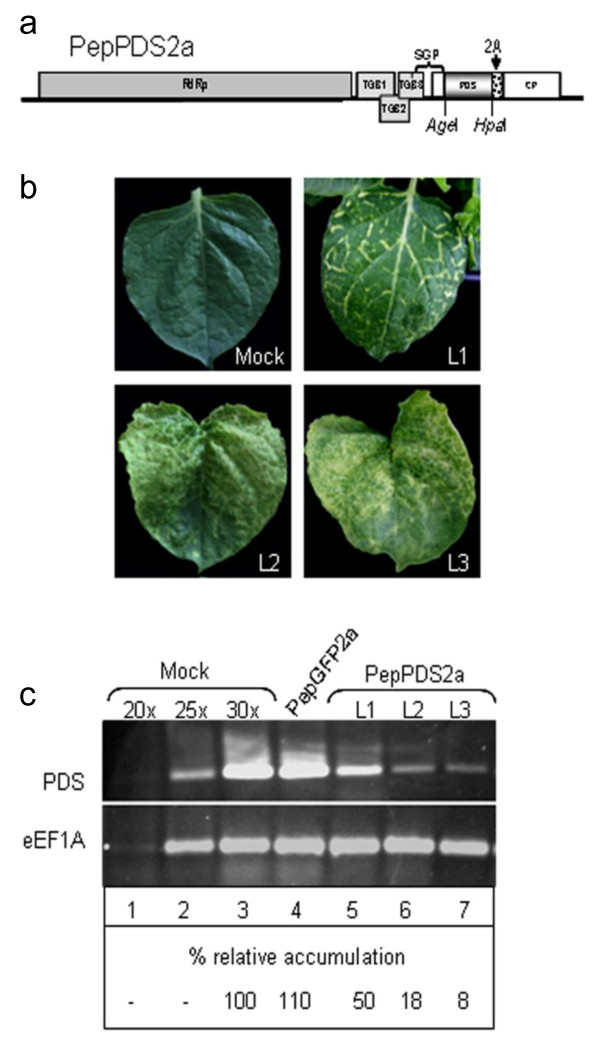
**PepMV based gene silencing vector**. (a) Schematic representation of PepPDS2a. (b) Representative leaves from *N. benthamiana *plants infected with the PepPDS2a vector showing different phenotypes of photobleaching (L1, L2 and L3). (c) Quantification of *pds *mRNA levels by semi-quantitative RT-PCR in plants inoculated with water (mock), pBPepGFP2a and pBPepPDS2a, as indicated. Lanes 1-3 correspond to mock-inoculated controls using 20, 25 and 30 amplification cycles. Thirty cycles were used for amplifications in lanes 4-7. L1, L2 and L3 correspond to the leaves with different phenotypes of photobleaching shown in the panel b. The relative (%) accumulation of *pds *mRNA in relation to mock-inoculated leaves is indicated below each lane.

Finally, we tested if PepMV could be used to generate a VIGS vector. A 335 bp fragment of the tomato phytoene desaturase (*pds*) gene was inserted into the PepGFP2a vector, replacing the *gfp *gene. The tomato *pds *fragment selected showed a sequence identity of 93% with the gene from *N. benthamiana*. Additionally, PepGFP2a was further modified to include additional cloning sites so that genes of interest could be inserted into AgeI and HpaI restriction sites. The resulting virus, PepPDS2a (Figure 7a), was used to infect *N. benthamiana *plants. All plants infiltrated with pBPepPDS2a began to display different photobleaching symptoms at 3-4 weeks post-infiltration (Figure 7b), attributed to the silencing of the endogenous *pds*. The silencing phenotype was first detected in the upper uninoculated leaves. It appeared first as a yellow-white coloration along the veins and then, as silencing progressed, yellow-white areas became visible in the leaves, covering with time almost the complete leaf.

To confirm VIGS at the molecular level, semi-quantitative RT-PCR was performed to compare the levels of *pds *transcripts present in leaves showing different photobleaching phenotypes (Figure 7c). The primers used for estimation of the *pds *mRNA levels were designed to anneal outside the region targeted for silencing, so that only the endogenous mRNA was probed. We used the eukaryotic elongation factor 1A (eEF1A) mRNA as a constitutively expressed internal control. Quantification of the signal brightness of DNA bands revealed that the *pds *mRNA was on average 80-90% less abundant in photobleached leaves of plants infected with PepPDS2a than in mock-inoculated leaves or in leaves infected with PepGFP2a (Figure 7c, lanes 3 and 4, respectively). In contrast, similar levels of eEF1A mRNA were found in all cases (Figure 7c). As expected, *pds *mRNA levels were smaller in leaves exhibiting more complete photobleaching (L2 and L3 in Figure 7b) than in leaves where the silencing was restricted to the veins (L1 in Figure 7b) (Figure 7c). Taken together, these findings suggest that the photobleaching symptoms in the PepPDS2a-infiltrated *N. benthamiana *plants can be attributed to silencing of the plant *pds *mRNA. These results indicate that PepMV can be used as a viral vector to silence genes in *N. benthamiana *plants.

## Discussion

In this work, two agroinfectious clones from two different PepMV genotypes (European and Chilean), able to initiate typical PepMV infections, were generated. We took advantage of these agroinfectious clones to develop a novel set of PepMV-based vectors. We investigated three key vector development strategies, namely replacement of the CP gene, duplication of the CP SGP and CP fusion using the FMDV 2A peptide. Although potexviruses are generally well-characterized, few studies have specifically focused on PepMV and little is known about the precise functions of its genes and proteins, and how these control its interactions with host plants. As part of our investigation, we have determined that the PepMV CP is required for cell-to-cell and long-distance movement, and we have gained insight into the minimal sequence required to act as a CP SGP.

We first sought to determine the relationship between CP expression and virus replication by constructing two PepMV mutants in which CP expression was abolished, in one case by a point mutation and in the other by the replacement of most of the CP ORF. Both mutants remained capable of replicating in protoplasts, confirming that the CP is not absolutely necessary for PepMV replication at the single-cell level. However, CP mutations in other potexviruses have been shown to reduce the amount of plus-sense RNA accumulating in protoplasts suggesting that, although the CP may not be essential for virus replication in single cells, it may stimulate this process [49,50,55]. Our results appear to rule out a direct and specific role of the CP, so we speculate that the point mutation in PepXL6agg may increase the rate of RNA degradation because of its untranslatable 3' end, as shown for many other plant mRNAs [56]. However, while our protoplast transfections were indicative, they were not strictly quantitative, so without additional experiments we cannot completely exclude the possibility that the CP may influence the accumulation of PepMV RNA at the single-cell level.

Agroinfiltration of *N. benthamiana *leaves with pBPepGFPΔCP showed that although CP-deficient PepMV mutants were competent for replication, they were unable to achieve cell-to-cell movement. This phenomenon is well documented in other potexviruses [49,50]. The movement of PepMV-CP mutants could be restored by providing CP in *trans *but there were only few infection foci, suggesting that PepGFPΔCP was able to initiate infection foci but with a low efficiency. Movement-deficient TMV expression vectors delivered into *N. benthamiana *leaves by agroinfiltration also initiated replication rather inefficiently, a phenomenon that was attributed to improper processing of the primary transcript in the plant nucleus [57]. The authors showed that the addition of introns and the removal of putative splicing sites increased the proportion of infected cells from 0.6% to 96%, and it is possible that the same strategy might improve the efficiency of PepMV-based vectors.

We constructed further PepMV vectors by duplicating the putative CP SGP and using this additional promoter to drive the GFP gene. Two vectors were prepared (pBPep501 and pBPep5.128) and both infected *N. benthamiana *leaves efficiently and were capable of systemic movement, resulting in strong GFP expression throughout the plant. However, this SGP duplication made the vector unstable and possibly prone to recombination; homologous recombination has been observed in many other viral vectors, resulting sooner or later in transgene elimination [33,38,58-61], although PVX-based vectors are remarkably stable [16]. Dragicci and Varrelamann [62] have shown that the frequency of transgene elimination in PVX vectors is proportional to the length of the homologous sequence available for template switching. Since our PepMV SGP is twice the size of the equivalent sequence in PVX vectors, that could be the reason for the observed instability [16]. The duplicated PepMV SGP contained all the *cis*-acting elements necessary for transgene expression. We attempted to increase the stability of the vector by removing potentially unnecessary downstream sequences from the duplicated SGP. Unfortunately, this size reduction reduced the level of virus RNA produced in infected plants, suggesting that the sequences between positions +12 and +36 are necessary for optimal SGP activity. Sequences within the CP ORF are also required for maximum sgRNA production in other viruses [63], usually by acting as enhancers [64,65].

Stability has been improved in other vectors with duplicated SGPs by using heterologous promoters to reduce the likelihood of recombination [59,63,65,66]. Accordingly, we generated a hybrid vector containing the SGPs from two distantly related PepMV isolates, PepMV-Sp13 and PepMV-Ps5. These sequences had an overall identity of 48%, although they contained a highly conserved 22-nt region at the 5' end including the potexvirus-specific octanucleotide motif discussed above. Although such heterologous sequences have increased the stability of TMV vectors [63], they had little impact in the case of PepMV: the hybrid vector suffered transgene loss at 7-10 dpi, similarly as with the duplicated SGP vectors. In agreement with this result, Nagy and Bujarski [67] showed that as little as 15 nt of sequence identity can promote homologous recombination in a brome mosaic virus vector. On the other hand, Dragicci and Varrelmann [62] provided evidence that RNA secondary structure was required to achieve precise homologous recombination. The PepMV CP SGP region potentially forms several stem-loops structures around the octanucleotide motif (data not shown) that may be involved in recombination events causing transgene elimination.

The FMDV 2A protease sequence has been used with success to overcome the problem of homologous recombination in viral vectors [34,68] and has been shown to work in the context of both PVX and CPMV [34,69]. We therefore developed a further vector (PepGFP2a) in which GFP is expressed fusioned with the CP, with an intervening 2A peptide. This strategy appeared to solve our instability problems observed with the other vectors. Although PepGFP2a spread more slowly than Pep501, systemic infection was nevertheless observed by 7 dpi, and GFP fluorescence persisted for several weeks. PepGFP2a was stably propagated through up to four serial passages, each passage resulting in a similar systemic infection characterized by long-lasting uniform fluorescence, with instability events only starting to occur at ~12 dpi in the third passage. This instability might result from the presence of a 36-nt duplication within the CP gene. Western blot analysis of infected plants showed that the fusion protein was expressed and correctly processed, confirming that the 2A protease was efficient in the context of PepMV infection (free CP was approximately tenfold more abundant than the GFP2aCP fusion protein showing that most of the fusion protein was fully processed). GFP accounted for 0.2-0.4 g/kg of fresh tissue, comparable to the yields reported for CPMV [32] and early versions of TMV [70]. These yields are already sufficient for most applications, although they are not as high as those reported for highly-developed systems [35,39,71,72]. We expect that further refinement of the vector (*e.g*., altering the viral sequence to improve mRNA processing after agroinoculation, improving inoculation methods and other parameters) [44,57] will lead to increased expression levels. The data presented here suggest that our system can be used for the expression of proteins that are at least as large as GFP. For the expression of larger proteins, systems based on "deconstructed" vectors (*e.g*., providing the CP in *trans*) could be explored [73].

The experiments carried out with PepGFP2a showed that PepMV viral vectors induce no or mild symptoms in the infected plants and that PepMV is distributed in most parts of the plants, infecting roots and flowers. These characteristics make PepMV an attractive system to be used as a tool for silencing genes in different tissues. Therefore, we constructed PepPDS2a and demonstrated that this vector is also suitable for VIGS experiments. Plants infected with PepPDS2a, carrying a fragment of the tomato *pds *gene, showed a clear bleaching phenotype visible at 21 - 28 dpi. Molecular analysis showed successful *pds *silencing in stronger bleached yellow-white leaves, whereas in the leaves with bleaching occurring only in their veins silencing was less efficient, showing a clear correlation between phenotypes and mRNA levels. The fact that silencing was not equally efficient in different leaves is probably due to an unequal distribution of the virus in the infected tissues. It is noteworthy that the silencing capacity of our PepMV-based VIGS vector is possibly higher, because the *pds *fragment (from tomato) inserted into the vector had only 93% of nucleotide identity with the homologous gene from *N. benthamiana*. Moreover, it has been demonstrated that the length and the position of the insert with respect to the full-length cDNA can affect silencing efficiency of a particular vector [74]. Therefore these aspects need to be taken into account in the design of new and specific PepMV-based VIGS vectors.

## Conclusions

In conclusion, we have demonstrated that PepMV can be used as an expression vector for recombinant protein expression in plants. Two distinct vector systems were envisaged, one based on an autonomous viral vector and another based on a "deconstructed" virus, where the CP is provided in *trans *[39,40,75]. Such vectors could serve for multiple purposes including the analysis of gene functions *in planta*, the expression of valuable recombinant proteins and the dissection of PepMV wild-type functions.

## Materials and methods

### PepMV agroinfectious clones

The full-length cDNAs of PepMV-Sp13 [13] and PepMV-Ps5 [3] were generated by RT-PCR using primers CE-42 (5'-CGC GGA TCC GGA AAA CAA AAT AAA TAA-3') or CE-292 (5'-GGG CCC GGA AAA CAA AAC ATA ACA C-3') and CE-43 (5'-(A)_20 _CCC GGG CCC GCG GTA CCC C-3'), corresponding to the 5' and 3' termini of the Sp13 and Ch2 genomic RNAs, respectively, and including the necessary restriction sites (underlined). First strand cDNAs were synthesized using primer CE-43 and the SuperScript First-Strand Synthesis System for RT-PCR (Invitrogen), and amplified with Pyrobest polymerase (Takara). The resulting DNA fragments were purified and inserted into the pTOPO-XL vector (Invitrogen), generating constructs pTPepXL6 and pTPepPs5. The pTPepXL6 construct was inserted between the BamHI and XmaI sites of pBIN61 [47] to generate pBPepXL6, whereas pTPepPs5 was inserted at the BamHI site of the same vector to generate pBPepPs5. Constructs for *in vitro *transcription using the T7 promoter were produced by amplifying the viral insert in PepXL6 with forward primer CE-484 (5'-AAT ACG ACT CAC TAT AGG GAA AAC AAA ATA AAT AA-3'; T7 promoter sequence underlined) and reverse primer CE-43.

### DNA constructs for PepMV mutants and vectors

All PepMV mutants and vectors were constructed using standard overlapping PCR and molecular cloning methods [76]. For the construction of pT7PepXL6agg, two overlapping DNA fragments were amplified in separate PCRs. In PCR 1, a DNA fragment containing the replicase and the TGB genes and the first 11 nt of the CP gene was amplified from pTPepXL6 using the primers CE-42 and CE-199 (5'-CAA TAA ACA ATC AgG CCT GAC AC-3'); the mutated nucleotide is shown in lower case within the context of the original ATG triplet, underlined). In PCR 2, a DNA fragment containing the CP gene and the 3' UTR was amplified with the primers CE-198 (reverse complement of CE-199) and CE-43. These overlapping fragments were mixed and amplified in a third PCR to produce a full-length DNA fragment using primers CE-484 and CE-43, complementary to the ends of the two initial fragments. The resulting PCR product was inserted into pTOPO-XL.

For the construction of pBPepGFPΔCP and pT7PepGFPΔCP, the same steps as above were carried out amplifying two overlapping DNA fragments in separate PCRs. In PCR 1, a DNA fragment covering the 3' end of TGB1 though to the 5' end of the CP gene was amplified using pTPepXL6 as the template and the primers Pep303 (5'-CGG AAT TGC AGG CAC TGG G-3') and CE-458 (5'-GTT GCT GCC ACT TCA AGT AGG AGT AAA GGA GAA GAA-3', including the first 18 nt of the GFP gene). CE-458 places the GFP ORF 36 nt downstream of the first CP codon. In PCR 2, a DNA fragment containing the GFP gene followed by the PepMV 3' UTR was amplified using primers CE-459 (reverse complement of CE-458) and CE-43. The overlapping products were mixed and amplified into a full-length DNA fragment using primers Pep303 and CE-43. The final PCR product was either inserted into pT7PepXL6agg using the XmnI and XmaI sites to produce pT7PepGFPΔCP, or inserted into pTPepXL6 using the same restriction sites followed by subcloning into the BamHI and XmaI sites of pBPepXL6 to obtain pBPepGFPΔCP.

For the construction of pBINCPep, a binary vector used to provide CP *in trans*, a DNA fragment corresponding to the PepMV CP gene was amplified using pTPepXL6 as the template and primers CE-356 (5'-CGC GGA TCC CAA TCA TGC CTG ACA C-3') and oligo d(T). The PCR product was introduced into the SmaI site of the binary vector pBIN61.

Overlapping PCR was also used to construct pBPep5.128. In PCR 1, a DNA fragment covering the 3' end of TGB1 through to the 3' end of the CP gene was amplified using pTPepXL6 as the template and primers Pep303 and CE-504 (5'-GAA AAC TTA ACC CGT TCC AAG TTA AAG TTC AGG GGG TG-3', including 18 nt of the TGB4 (from 5563 to 5581), considered as the 5' end of the putative CP SGP). In PCR 2, a cDNA fragment containing the GFP gene fused to the PepMV-3' UTR was amplified using primers CE-503 (reverse complement of CE-504) and CE-43. These PCR products were mixed and amplified in the third PCR with primers Pep303 and CE43. The resulting fragment was introduced into the XmnI and XmaI sites of pTPepXL6, and a BamHI/XmaI fragment from this construct was introduced into the BamHI and XmaI sites of the binary vector pBPepXL6. For the construction of pBPep501 by overlapping PCR, the same templates were used but in a different order. In this case, PepGFPΔCP was used as a template in PCR 1 and pTPepXL6 in PCR 2, using the complementary primers CE-502 (5'- GAT GAA CTA TAC AAA TAA CTT GGA ACG GGT TAA GTT TTC-3') and CE-501 (5'- GAT GAA CTA TAC AAA TAA CTT GGA ACG GGT TAA GTT TTC-3') corresponding to the last 18 nt of the GFP gene and the first 18 nt of TGB4 (from 5563 to 5581). The resulting fragment was transferred to pTPepXL6 and then to pBPepXL6 as described above.

For the construction of pBPep352 and pBPep640, the procedures and templates were the same as those used to produce pBPep501. Two consecutive sets of overlapping PCR were performed. The first was similar to that used to prepare pBPepGFPΔCP but with different complementary primers. For pBPep352, the primers were CE-352 (5'-CCT GAC ACA ACA CCT GTT CCG CGG ATG AGT AAA GGA-3') and CE-350 (reverse complement of CE-352). For pBPep640, the primers were CE-640 (5'-AAC AAT CAT GCC TGA CAC AAT GAG TAA AGG AGA AGA-3') and CE-641 (reverse complement of CE-641). The second overlapping PCR and subsequent cloning steps followed were exactly as described for pBPep5.128.

Two consecutive sets of overlapping PCRs were also required for the construction of pBPep505. The first of overlapping PCRs was used to generate the SGP_Ps5_-GFP-3' UTR fragment. In PCR 1, a DNA fragment containing the putative SGP_Ps5 _was amplified using pTPepPs5 as the template and primers CE-497 (5'-GCA ATA AAC TTC TCC CCT TGG AAC GGG TTA AGT-3', corresponding to 18 nt of PepMV-Sp13 TGB4 (from 5545 to 5563) and 18 nt of PepMV-Ps5 TGB4 (from 5553 to 5571)) and CE-498 (5'-GCT TCT AAC CCA TCA GAT ATG AGT AAA GGA GAA GAA-3', corresponding to 18 nt of the CP gene (from 5651 to 5669) and 18 nt of the GFP gene). In PCR 2, a DNA fragment comprising GFP fused to the PepMV-3' UTR was amplified using primers CE-499 (the reverse complement of CE-498) and CE-43. The overlapping products were joined in a third PCR using primers CE-497 and CE-43. The second set of overlapping PCRs was as described for the construction of pBPep5.128 although with the SGP_Ps5_-GFP-3' UTR fragment used instead of pBPepGFPΔCP in PCR 2. The subsequent cloning steps were the same as those used in the construction of pBPep5.128.

Two consecutive sets of overlapping PCRs were also used for the construction of PepGFP2a (pBPepGFP2a). In PCR 1, a DNA fragment covering the 3' end of TGB1 through to the 5' end of the CP gene was amplified using pTPepXL6 as the template and primers Pep303 and CE-458. In PCR 2, a DNA fragment containing the GFP gene and 2A sequence was amplified using CE-459 (the reverse complement of CE-458) and CE-665 (5'-GAG TCC AAC CCT GGG CCT GAC ACA ACA CCT GTT-3', corresponding to last 18 nucleotides of 2A sequence and nt 4-21 of the CP). Because 2A-mediated processing requires a proline to follow the 2A sequence (NFDLLKLAGDVESNPG), it was inserted between the last codon of the GFP gene and the second codon of the CP gene, which specifies a proline residue. The products were mixed and amplified to obtain the full-length DNA using primers Pep303 and CE-665. The second set of overlapping PCRs was similar to those used in the construction of Pep501, although the fragment obtained with primers Pep303 and CE-665 was used as the template in PCR 1. and in PCR 2, a DNA fragment containing the CP gene and 3' UTR was amplified using primers CE-664 (the reverse complement of CE-665) and CE-43. The subsequent cloning steps were as described for the construction of pBPep5.128.

The design of pBPepPDS2a was basically the same as for pBPepGFP2a but in this case we introduced a portion of the tomato *pds *sequence [from 962 to 1319; GeneBanK 544073] flanked by sites AgeI and HpaI instead of the *gfp *sequence (Figure 7a). For the construction of pBPepPDS2a, a DNA fragment reaching from the 3' end of TGB1 until the 3' UTR with a XmaI site inmediatly after the poly(A) tail, and including the tomato *pds *sequence in-between as mentioned before, was synthesized by GeneScript (New Jersey, USA). This fragment was digested with XmnI and XmaI and introduced into the same sites of pTPepXL6, and further subcloned into the binary vector pBIN61 using the BamHI/XmaI restriction sites, resulting in pBPepPDS2a.

### In vitro transcription and protoplast transfection

Plasmids pT7PepXL6, pT7PepXL6agg and pT7PepGFPΔCP were linearized with *Kpn*I and transcribed *in vitro *in the presence of cap analog (m^7^G^5^pppNp) (Promega) using RiboMAX™ Large Scale RNA Production Systems (Promega) according to the manufacturer's instructions. *N. benthamiana *plants (4-5 weeks old), grown in a growth chamber (16-h photoperiod, 22°C), were used for preparation of mesophyll protoplasts as described by Weston and Turner [77]. Approximately 10^6 ^protoplasts were electroporated with 10 μg of *in vitro *transcribed RNA. The protoplasts were incubated in the dark at 25°C and harvested after 24 h for RNA extraction.

### Inoculation and fluorescence visualization

*Agrobacterium tumefaciens *strain C58C1 was transformed with the different PepMV constructs cloned in binary vector pBIN61 by electroporation. Overnight cultures (3 ml) were centrifuged at 2,500 g for 10 min. The pellet was resuspended in the same volume of 10 mM MES (pH 5.5), 10 mM MgSO_4 _and 100 μM acetosyringone. Leaves of *N. benthamiana *plants from the greenhouse were infiltrated using a syringe without a needle. For *trans*-complementation analysis, *A. tumefaciens *cultures were mixed in a 1:1 ratio. If P19 was used in the experiments, *A. tumefaciens *cultures containing the pBP19 vector were mixed with those containing the PepMV constructs at a 1:3 ratio. Mechanical inoculation was carried out by homogenizing leaf tissue in 30 mM sodium phosphate, pH 8.0, followed by rubbing 3-week-old carborundum-dusted *N. benthamiana *leaves with the extract. *N. benthamiana *plants (4-5 weeks old) were grown in a growth chamber (16-h photoperiod, 25°C for GFP expression or 22°C to induce silencing). Leaves were viewed under UV light (365 nm) provided by a handheld lamp (Blak Ray B100-AP lamp, UV products, Upland, CA 91786, USA). For confocal imaging, 0.5 cm^2 ^squares of leaf tissue were prepared, mounted in water and imaged using a Leica TCS SP2 (Leica Microsystems, Germany) inverted confocal laser scanning microscope. The fluorescence intensity was measure using Qwin V3 (Leica) image analysis software.

### RNA and protein analysis

RNA was extracted from protoplasts or leaf tissue using TRI-Reagent (Sigma Chemical Co., St. Louis, MO) and separated in agarose gels containing formaldehyde. The RNA was transferred to positively charged nylon membranes (Roche, Indianapolis, IN, USA), which were hybridized with digoxigenin-labeled probes (prepared by in vitro transcription) specific for PepMV replicase, PepMV CP or GFP [76]. Chemiluminescent detection was carried out using the reagents and protocols supplied in the DIG-labeling and detection kit (Roche Diagnostics). For protein extractions, 100 mg samples of fluorescent *N. benthamiana *leaves were ground in 200 μl protein extraction buffer (0.1 M Tris, pH 8.0, 0.125 mM 2-mercaptoethanol, 200 mM PMSF, 10% glycerol). Total soluble protein content was determined using the Bradford assay. Crude extracts were mixed with 4 × loading buffer and separated by SDS-PAGE followed by staining with Coomassie Brilliant Blue or by electrotransfer to nitrocellulose membranes. Blots were probed with monoclonal antibodies raised in rabbits against PepMV CP (AC Diagnostics, Fayetteville, USA) or in mouse against GFP (Clontech, Palo Alto, CA, USA) followed by detection with anti-rabbit or anti-mouse immunoglobulin G (IgG) coupled to horseradish peroxidase (Promega) and chemiluminescence (ECL Western Blotting and Protein Analysis System, Amersham Biosciences). The image was taken with the GeneSnap software on a G:BOX (Syngene, Cambrige, UK) and signal intensity of DNA bands quantified using the Gene Tools software (Syngene, Cambrige, UK).

### Semiquantitative RT-PCR analysis

For semiquantitative RT-PCR analysis, leaves expressing different bleaching phenotypes were harvested at 5 and 6 weeks post-infiltration. Total RNA was extracted using Tri-Reagent and samples were treated with RNase-free Dnase I (Roche) prior to reverse-transcription with the One-Step RT-PCR kit (Qiagen, Valencia, CA, USA) according to the supplier's instructions. Primers CE-787 (5'-TTT CCA AAA GTT GGA GAA GC-3') and CE-788 (5'-TCA TGT TGT CAA AAC CCC AA-3') used to amplify the *pds *mRNA, annealed outside the region targeted for silencing. Semiquantitative RT-PCR was performed as described by Wang *et al. *[78] using primers CE-780F (5'-ACA AAC CCC TCC GTC TTC CAC-3') and CE-781 (5'-CCC TAC TGG TTT GAC AAC TGA-3') to amplify eEF1A mRNA as internal, constitutively expressed control. RT-PCR reactions, in a final volume of 25 μl, contained 250 ng of RNA and 0.6 μM of each pair of primers. After 25 and 30 cycles, the PCR-generated fragments were analyzed in 1.5% agarose gels, and the intensity of the amplified fragments was quantified using GeneSnap sofware tools.

## Competing interests

The authors declare that they have no competing interests.

## Authors' contributions

RNS designed the experiments, carried out most of the experimental work, wrote the manuscript and co-participated in the conception of the work. PG developed the agroinfectious clone for PepMV-Ps5. VT co-participated in the conception of the work and supervised the laboratory work for the generation of the DNA constructs. MAA is the principal investigator, conceived the project and supervised the experiments and the writing of the manuscript. All authors read and approved the final manuscript.
